# Do Parentese Prosody and Fathers' Involvement in Interacting Facilitate Social Interaction in Infants Who Later Develop Autism?

**DOI:** 10.1371/journal.pone.0061402

**Published:** 2013-05-01

**Authors:** David Cohen, Raquel S. Cassel, Catherine Saint-Georges, Ammar Mahdhaoui, Marie-Christine Laznik, Fabio Apicella, Pietro Muratori, Sandra Maestro, Filippo Muratori, Mohamed Chetouani

**Affiliations:** 1 Department of Child and Adolescent Psychiatry, Assistance Publique-Hôpitaux de Paris, Groupe Hospitalier Pitié-Salpêtrière, Université Pierre et Marie Curie, Paris, France; 2 Institut des Systèmes Intelligents et de Robotique, Centre National de la Recherche Scientifique UMR 7222, Université Pierre et Marie Curie, Paris, France; 3 Department of Child and Adolescent Psychiatry, Association Santé Mentale du 13^ème^, Paris, France; 4 Division of Child Neurology and Psychiatry, University of Pisa, Stella Maris Scientific Institute, Calambrone, Italy; UCLA, United States of America

## Abstract

**Background:**

Whether development of autism impacts the interactive process between an infant and his/her parents remains an unexplored issue.

**Methodology and Principal Findings:**

Using computational analysis taking into account synchronic behaviors and emotional prosody (parentese), we assessed the course of infants' responses to parents' type of speech in home movies from typically developing (TD) infants and infants who will subsequently develop autism aged less than 18 months. Our findings indicate: that parentese was significantly associated with infant responses to parental vocalizations involving orientation towards other people and with infant receptive behaviours; that parents of infants developing autism displayed more intense solicitations that were rich in parentese; that fathers of infants developing autism spoke to their infants more than fathers of TD infants; and that fathers' vocalizations were significantly associated with intersubjective responses and active behaviours in infants who subsequently developed autism.

**Conclusion:**

The parents of infants who will later develop autism change their interactive pattern of behaviour by both increasing parentese and father's involvement in interacting with infants; both are significantly associated with infant's social responses. We stress the possible therapeutic implications of these findings and its implication for Dean Falk's theory regarding pre-linguistic evolution in early hominins.

## Introduction

Autism spectrum disorders (ASD) are developmental disorders that are characterised by the presence of symptoms in 3 domains: (i) abnormalities in reciprocal social interactions; (ii) abnormalities in patterns of communication; and (iii) a repetitive repertoire of behaviours and interests. ASD are heterogeneous in terms of clinical manifestations and course, but children's difficulties with adequate socialization or communication are at the core of the pathologic developmental course. The most typical form of ASD is autism that presents with symptoms in all 3 domains and has an early age of onset (before 3 years). Symptoms, such as speech delay and stereotyped behaviour, are often evident between 18 and 36 months, but these clearly are not the initial manifestations of autism [1]. Studies using early home videos [2], [3] parental interviews [4] and prospective assessment of siblings of children with ASD [5], [6] have revealed atypical developmental tendencies in infants who were later diagnosed with ASD. The first signs are abnormalities with eye contact, imitation, disengagement, joint attention, orienting to name, and body language. These behaviours constitute important precursors of later-developing symptoms; however, whether these first signs impact the interactive process between an infant and their parents and whether they influence the development of the infant himself remain two complex and unexplored issues.

Human learning and cultural evolution are supported by paradoxical biological adaptation. We are born immature; yet, immaturity has value: “*Delaying maturation of cerebral cortex allows initial learning to influence the neural architecture in ways that support later, more complex learning*” [7]. Early learning appears to be computational [8] and to be based on perceptual-action mapping [7]. Learning is also social [9] and supported by skills present in infancy: imitation, shared attention and empathic understanding [7]. The whole social system which contributes to interactional synchrony and attunement may be disrupted (as explained above) in infant who will subsequently develop autism.

It is likely that an atypical social trajectory in the infant would affect parents' interactive patterns. However, very few studies have addressed the importance of infant-caregiver synchrony/reciprocity in early interactions involving infants who will subsequently develop autism. Temporally, the interactive nature of human communication implies that a message a_i_ produced by A impacts B who, in return, produces message b_i_ and so on, indicating that some form of reciprocity occurs between partners A and B [10]. Synchrony is difficult to define and delimit. Numerous terms have been used to describe the interdependence of dyadic partners' behaviours (mimicry, social resonance, coordination, synchrony, attunement, chameleon effect, etc.). Here, we define synchrony as the dynamic and reciprocal adaptation of the temporal structure of behaviours between interactive partners [11]. In typically developing children, the quality of social interaction depends on an active dialogue between the parent and the infant based on the infant's desire to be social and the parent's capacity to be attuned [12], [13]. Numerous studies have been emphasising the importance of synchrony and the co-modality [14]. In a previous study based on home movies (HM), we showed that when studying interactive patterns with computational methods to take into account synchrony between partners, (i) deviant autistic behaviours appeared before 12 months; (ii) parents seemed to feel weaker interactive responsiveness and mainly weaker initiative from their infants; and (iii) parents increasingly tried to supply soliciting behaviours and touching [15]. It is likely that these modifications of interactive patterns implicate numerous co-influences due to the reciprocal nature of these processes.

A special type of speech directed towards infants, called ‘parentese’, is characterised by higher pitch, slower tempo, and exaggerated intonation contours [16]; it appears to be universal and to play an important role in social interaction and language development. Parentese has been shown to be present in mothers, but also in fathers and other caregivers when addressing an infant [17]. This particular prosody may be responsible for attracting an infant's attention, conveying emotional affect and providing language-specific phonological information [16]. Parentese was found to depend on the quality of the infant's responsiveness, suggesting that infants are actively involved in the course of parentese [18], [19]. Also, voice-sensitive brain regions are already specialized and modulated by emotional prosody by the age of 7 months. This raise the possibility that the critical neurodevelopmental processes underlying impaired voice processing in autism might occur early during infancy [20]. Finally, parentese may not only be crucial during early development but also for species evolution. Falk proposed parentese to be a key adaptive human skill during the transition between late australopithecines and early Homo. This hypothesis is based on the premise that hominin mothers who vigilantly attended to infants were strongly selected for and that such mothers had the ability to modify their vocalisations to control infants who were too immature at birth to grasp their mothers as chimps do (see the Discussion section for further exploration of this topic) [19].

Therefore, the study of parentese in early interactions with infants who will later be diagnosed with autism may provide cues for understanding the disruption of social and communicative skills in children with autism. We postulated that if learning and development depend on normal social interest in people and the signals they produce, children with autism, who lack social interest, may be at a cumulative disadvantage in development and language learning. Their poor response to parental solicitation may impair both parental solicitation and parentese production over time. As a consequence, this impairment will reinforce social withdrawal and language acquisition delay [21], [22].

In this study, we used a computerised algorithm created for the detection of parentese based on acoustic components [21] and focused on parents' type of affective speech and infants' responses simultaneously using HM from two groups: typically developing (TD) children and children who subsequently developed autism (AD). We tested the following hypotheses: (H1) as parentese amplification is bidirectional and children with AD lack social interest, parentese towards children with AD should decrease over time; this decrease will likely be greater than that observed in parentese towards TD children; and (H2) AD infants should be capable of interacting and responding to parentese at the start of life. This hypothesis was based on findings from HM studies and parental interviews showing that the very early behaviours of infants who later develop autism do not differ dramatically from typical developing controls [1]–[5]. A previous exploratory case study, comparing a TD child and an AD child, supported this hypothesis: the course of an infant's response to CG vocalization differed according to the type of speech (motherese vs. other speech) and child status (TD vs. AD). Mothers spent more time interacting with infants, and fathers appeared to interact with their child preferentially between 12 and 18 months in the TD boy [22]. This prompted us to distinguish between mothers' and fathers' interactive patterns, as such patterns may dramatically differ in terms of parentese use and time course.

## Materials and Methods

### Overview

**Figure 1:**
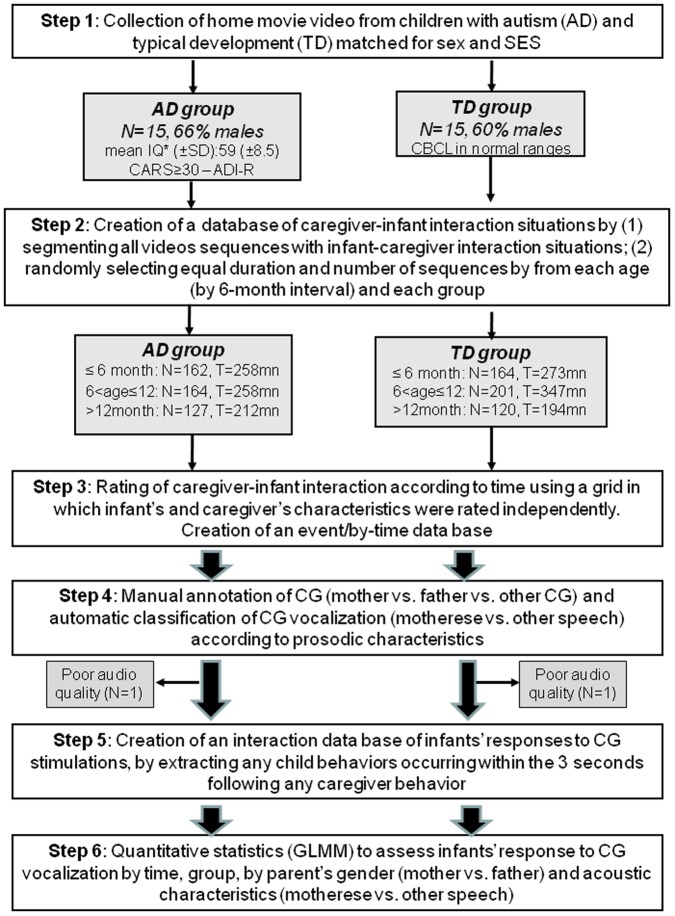
Diagram flow of the study. TD = Typical Development; AD = Autism Disorder; SES = Socio Economic Status; IQ = Intellectual quotient; CARS = Children Autism Rating Scale; CBCL = Child Behavior Check List; SD = Standard Deviation; CG = Care Giver; GLMM = Generalised Linear Mixed Model; *IQ in children with AD was based on the Griffiths Mental Developmental Scale or the Wechsler Intelligence Scale.

The flow diagram of the study is summarised in Figure 1. Thirty children were randomly selected from the Pisa HM database with the following criteria: 15 will be diagnosed with AD, and 15 will develop normally (step 1). All scenes showing a situation in which social interaction could occur (i.e., all scenes with an infant and an adult) were extracted and, if necessary, segmented into short sequences to be scored (step 2). Raters blind to diagnosis rated parents and infant behaviours independently within each interaction sequence according to a grid with a specific part for each partner (step 3). To distinguish parents' style of affective speech according to their emotional and prosodic characteristics, we classified all parents vocalisations as parentese or other speech (i.e., non-parentese) using an automatic algorithm [21]. At this stage, we lost one child per group because of poor HM audio quality. Additionally, to distinguish mother vs. father vs. other CG contributions to CG to infant interactions, we annotated CG accordingly (step 4). To study specifically how infants responded to parents' stimulations, we selected any child behaviors occurring within the 3 seconds following any parents' behavior (step 5). A generalised linear mixed model (GLMM) was performed to assess and compare infants' response to parents' vocalisations during interactive patterns by time and by group (step 6).

### Participants (Step 1)

We studied home movies (HM) from the first 18 months of life of two groups of children that were match for age and sex. The first group was composed of 15 children (M/F: 10/5) with a diagnosis of Autistic Disorder (AD) according to the DSM-IV criteria. In addition, we used the Autism Diagnostic Interview-Revised (ADI-R) for diagnosis [23] and the Childhood Autism Rating Scale (CARS) to assess the severity of autistic symptoms [24]. All cases with a CARS total-scores below 30 and with Pervasive Developmental Disorders Not Otherwise Specified were excluded. The absence of an identified genetic or metabolic disorder and of a severe sensory or motor impairment were verified for all children. The AD group was composed of children with an early onset autism without any history of regression: it means that all children displayed, from the beginning, the autistic symptoms constellation rated through the Behavioral Summarized Evaluation applied to the home movies of the first year of life (see Muratori et al., 2010 [25]). The children were recruited among those referred from multiple community sources to the the Scientific Institute “IRCCS Stella Maris”, a suburban university hospital providing tertiary care to patients of all socioeconomic levels. The study has been approved by the Ethical Committee of the Stella Maris Institute/University of Pisa, Italy. The Pisa HM data base includes children from biparental families with either typical or AD matched for gender and socio-economic status (all medium or good). Each child included in the Pisa HM data base had at least one scene from each of the first 3 semesters of life showing a situation in which social interaction could occur. Also, HM run for a minimum of 10 minutes for each of the first 3 semesters of life. The control group was recruited among children attending a local kindergarten attending a local kindergarten. It included 15 typically developing children (M/F ratio: 9/6) confirmed by non-pathological scores on the Child Behavior Check List [26]. After the automatic algorithm affective speech classification (step 4), the experimental group included 14 children (M/F ratio: 10/4) with a diagnosis of autism disorder (AD) without any sign of regression confirmed by the ADI-R. All AD children had a Childhood Autism Rating Scale (CARS) score >30. Mean Intellectual Quotients (SD) were 72.86 (8.5). Detailed clinical data for each child are given in Table S1 (available online).

### Extraction of parent-infant interactions (Step 2)

An editor, blind to child diagnoses, selected all segments running for at least 40 seconds where the infant was visible and could be involved in human interaction from among the HMs of each child. Sequences with more than one adult interacting with the infant (aside from the adult holding the video camera) were excluded. For each infant, the sequences were organised in three periods of 6 months of age (≤6 month; 6–12 months; >12 months) labelled semester 1 (S1), semester 2 (S2) and semester 3 (S3), respectively. Sequences were randomly selected by group and by semester. Preliminary t-test analysis showed that the selected video material was comparable across groups and for each age range in length and number of human interactions [25]. The number of scenes per infant is given in second column of Table S2 (available online).

### Computer-based coding system (Step 3)

The Observer 4.0® was configured for the application of the Infant Caregiver Behavior Scale (ICBS) to the video file. The ICBS (Annex S1 available online) is composed of items assessing the ability of the infant to engage in interactions and items describing CG solicitation for attention or stimulation of the infant. All target behaviors were described as Events which take an instant of time. CG regulation up and CG regulation down were described as events that take a period of time and have a distinct start and end.

Four coders were trained to use the computer-based coding system until they achieved a satisfactory agreement (Cohen's Kappa ≥0.7). The interactions derived from the HM of the two groups of children (AD and TD) were mixed, and each one was rated by one trained coder who was blind to which group the child belonged. For a continuous verification of inter-rater agreement, 25% of interactions were randomised and rated by two coders independently. The final inter-rater reliability, calculated directly by the Observer, showed a satisfactory Cohen-κ mean value ranging from 0.75 to 0.77.

### Studying parental and parentese contributions to parents infant interactions (Step 4)

To extract the caregiver's vocalisations from the video segments, we performed a manual segmentation of parents' vocalisations with The Observer 4.0®. We used Sound Forge 9.0 to extract the vocal segments from each video sequence. Vocal segments that were presented with poor audio quality or were imperceptible to the ear were excluded. At this stage, we lost one child per group because of poor HM audio quality. Manual segmentation provided us with a database of speech segments separated by cases (AD, TD) and by semester (S1, S2 and S3). In addition, manual segmentation allowed us to identify the following: the number/frequency of vocal segments, the duration of segments in each sequence, and who was speaking to the child (i.e., mother, father, other CG). Detailed data regarding the number of parent, mother, and father vocalizations as well as infant responses per infant are given in Table S2.

The segments of speech were analysed using a computerised classifier for categorisation as ‘parentese’ or other speech'. Classification according to acoustic components was required, as the interaction database included only CG vocalisations addressed to the infant. Because the "manual" study of acoustic components of the voice takes a very long time [27] and only allows for the study of very short voice segments, the use of such an algorithm made it possible to conduct an extensive study of HMs based on their acoustic characteristics.

The whole system was described by Mahdhaoui et al. [21]. Here, we briefly describe the key components of this automatic system. Using classification techniques that are often used in speech and speaker recognition (GMM, Gaussian Mixture Model, and K-nn, k-nearest neighbours), we developed a parentese detection system and first tested it on the mode dependence of the speaker. The fusion of features and classifiers was also investigated. Given that HM are not recorded by professionals and often contain adverse conditions (e.g., with regard to noise, the camera, or microphones), acoustic segmentation of HM shows that segmental features play a major role in robustness [28]. Consequently, the utterances were characterised by both segmental (Mel Frequency Cepstrum Coefficients) and supra-segmental (e.g., statistics with regard to fundamental frequency, energy, and duration) features. We showed that segmental features alone contained very useful information for discrimination between parentese and other speech, and the combination of segmental features with supra-segmental ones outperformed the supra-segmental features. However, according to our detection results, prosodic features were also very promising. Based on the previous two conclusions, we combined classifiers that used segmental features with classifiers that used supra-segmental features and found that this combination improved the performance of our parentese detector considerably. In its most effective configuration, the novel detector used only the GMM classifier for both segmental and supra-segmental features (M, number of Gaussians for the GMM Classifier: M = 12 and M = 15, respectively, and λ = weighting coefficient used in the equation fusion: λ = 0.4). Performance was as follow: accuracy = 87.5% (95%CI = 82.91–92.08%); Positive Predictive Value, PPV = 88.47% (95%CI = 83.03–95.18%); Negative Predictive Value, NPV = 86.41% (95%CI = 79.4–92.88%) [21]. We also investigated the performance of the detector with these fusion parameters in detecting parentese versus other speech (non- parentese) within a second set of 200 utterances (100 parentese vs. 100 other speech) that were blindly validated by two psycholinguists extracted from 10 randomly-selected home videos with 12 independent speakers (all mothers). Performances under speaker-independent conditions were as follows: accuracy = 82% (95%CI = 73.87–89.58%); PPV = 86.36% (95%CI = 66.52–89.48%); NPV = 77.55% (95%CI = 76.73–96.6%) [21]. For the purpose of the current study, which also aimed to explore parentese in fathers, we analysed 100 sequences from fathers (50 parentese vs. 50 other speech) that were blindly validated by two psycholinguists. The system's performance was good: accuracy = 74% (95%CI = 64.27–82.26%); PPV = 71.43% (95%CI = 57.79–82.70%); NPV = 77.27% (95%CI = 62.16–88.53%). This level of prediction made it suitable for further studies of home videos.

For the interaction analysis, we first fused video coding and acoustic analysis. The computer-based coding system (The Observer) provided “.txt” files with the description of ICBS behaviours based on time. Vocalisations analysed by the computerised system provided two new tags: parentese vocalisation or other speech. These tags were reintegrated to the txt file as CG “state events”. To summarise, CGinfant interactive behaviours could now be examined by type of vocalisation (parentese vs. other speech), semester (S1, S2 or S3) and speaker (mother vs. father vs. other CG) in terms of frequency (number of events) and duration of CG vocalisations (in seconds).

### Creation of the interaction database (step 5)

We first created an interaction data base by extracting all child behaviors occurring within the 3 seconds following any caregiver behavior (including events that occur within the same second). The 3 second window was based on available literature on synchrony and previous work [14], [15]. Extraction was performed using a Linux-based script. The sequence of n interactive patterns is termed n-gram as usually performed in natural language processing or gene analysis. In this study, we only focused on bi-gram modelling. Given the large number of possible types of interaction ([CG item × infant item]), and the low frequency of several items in the data base, we created five CG meta-behaviors (Vocal solicitation, Touching, Gestural solicitation, Regulation up, Regulation down) and six infant meta-behaviors (Behavior with object and 5 types of Behaviors with people: Vocalizations, Inter-subjective behavior, Seeking people, Receptive to people, Orienting towards people,) by grouping ICBS items. Meta behaviors are shown in the left column of ICBS Grid and are based on previous studies [15], [25] (Annex 1). Then, we repeated the process of extraction to obtain finally, for each standard situation, all sequences of CG meta-behavior and infant meta-behavior occurring within a 3-second time window.

### Quantitative statistics (Step 6)

Following this multivariate analysis with four fixed factors, we conducted two post-hoc multivariate analyses (also using GLMM and infant response following a parental vocalization as the dependent variable). The first post-hoc analysis investigated Semester, Speaker and Vocalisation Type as independent variables within the same Group and the second with just Speaker and Vocalisation Type as independent variables within each Group and Semester category, still considering subjects as a random effect. We used a two-tailed threshold of significance for each calculation of p, and p = .05 was the level of significance.

## Results

### Parentese in the HM by group

**Figure 2:**
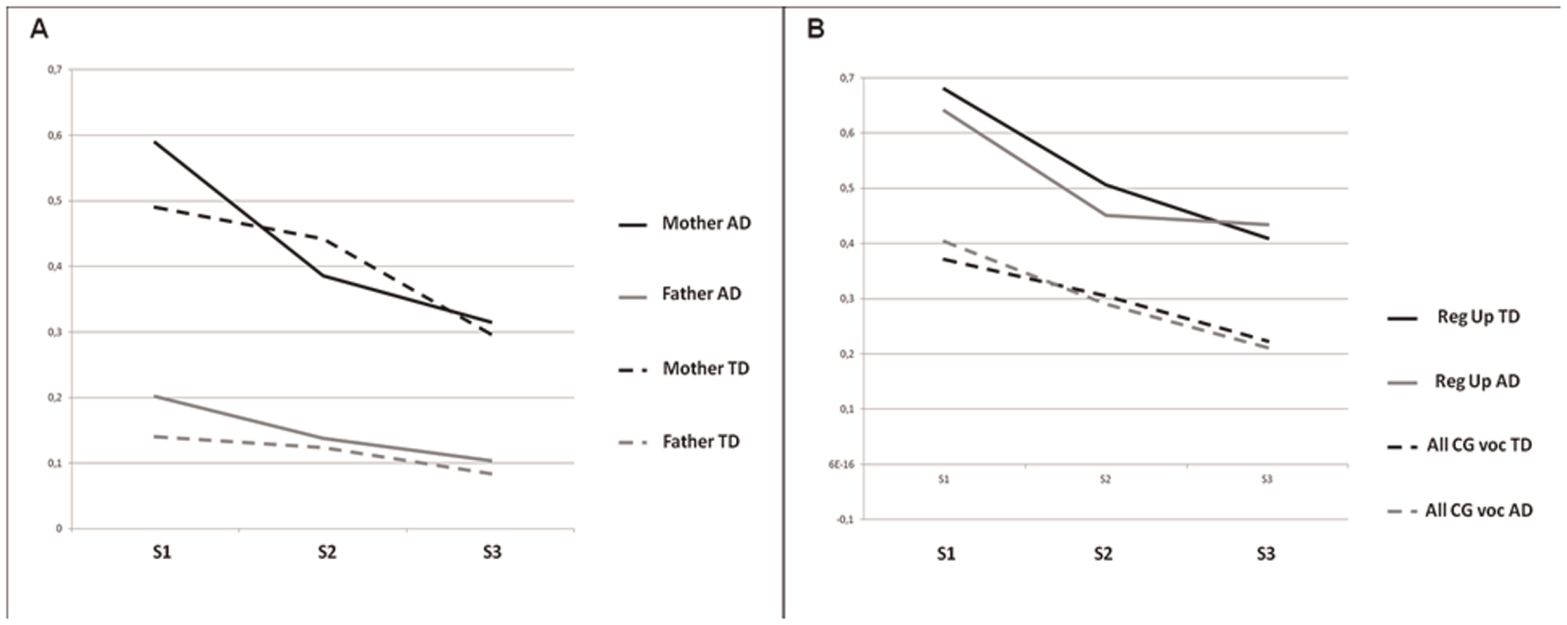
Proportion of parentese based on acoustic characteristics in mothers' and fathers' vocalisation of TD infants or infants with AD (2A); in caregiver Regulation Up and all vocalisation whether the infant had TD or AD (2B). TD = Typical Development; AD = Autism Disorder; CG = Care Giver; Reg Up = Regulation Up; All voc = All vocalisations.

We first explored, as a whole, before the interactive database extraction (step 5), the proportion of parentese in parents' vocalisation and in parents' soliciting behaviour (called Regulation Up in the ICBS) by group (figure 2A). As shown in figure 2A, we did not find any differences between groups for parents' vocalisation. The proportion of parentese was higher in mother vocalisations (40 to 60% of parentese) than father vocalisations (15 to 20% of parentese). Use of parentese among mothers showed the same decrease over time in both groups (β = −0.51; standard error = 0.097; p = <10^−6^; β = −0.55; std error = 0.11; p = <10^−6^ for S2 vs. S1, and S3 vs. S2, respectively). Parentese among fathers significantly decreased between S1 and S2 (β = −0.4; std error = 0.165; p = 0.015) but not between S2 and S3 (β = −0.29; std error = 0.178; p = 0.108) in both groups. Fathers of AD children tended to use more parentese than those of TD children (β = 0.6502; std error = 0.3355; p = 0.052) and also assumed a greater part in vocalizations (either using parentese or other speech) addressed to infants (β = 0.794; std error = 0.3752; p = 0.03). In other words, fathers of AD children spoke to their infants more than fathers of TD children regardless of whether they used affective speech. Regarding parents Regulation Up (figure 2B), we found that these parents soliciting behaviors were “full” of parentese, nearly doubling the proportion of parentese found in all vocalizations in both groups and at all 3 semesters (40 to 70% vs. 20 to 40% of parentese in parents Regulation Up and parents all vocalizations, respectively).

### Infant response to parents' vocalisation by time, group, parent's gender and acoustic characteristics

Table 1 shows the number of parental vocalizations and the overall number of infant responses following parental vocalizations as well as the number of infant responses according to subtype (e.g., towards an object in comparison to towards people) for each semester. The GLMM analysis is summarised in table 2. Considering any infant meta-behaviors that were a response to each parent's vocalization within a 3-second time window (parentsinfant interaction), the probability of infant responses increased significantly between the first and the second semester and then remained stable in quantity. However, they significantly changed in terms of quality of response: between S2 and S3, the probability of infant responses to parental vocalizations that involved orientation “towards an object” increased, whereas infant responses to parental vocalizations that involved orientation “towards people” decreased. Finally, the probability of infant intersubjective responses showed a continuous increase from S1 to S3. When considering the AD compared with TD group, we found that infants who later developed AD had fewer overall responses, specifically fewer responses to parental vocalizations that involved orientation “towards people” and less intersubjective behaviour. Post-hoc analyses conducted by semester showed that this lower rate of response to parents' vocalization was significant for AD infants as early as the first semester (β = −0.52; std error = 0.2613; p = 0.04).

**Table 1:** Number of parental vocalizations and number of total infant responses and infant responses according to subtype per semester.

	Parental vocalizations	Total infant responses	2 types of infant responses		5 categories of responses toward people				
			Toward object	Toward people	Receptiveresponses	Expressiveresponses	Active responses	Exploratory responses	Inter-subjective responses
*Infantwhowilllaterdevelopautism (N = 14)*									
S1	652	315	44	271	88	128	13	32	10
S2	983	619	116	503	121	217	35	88	42
S3	672	418	127	291	44	102	49	42	54
*Infantwithtypicaldevelopment (N = 14)*									
S1	950	578	115	463	126	229	27	57	24
S2	1170	824	167	657	154	253	60	76	114
S3	769	530	146	384	32	155	30	31	136

S_i_ =  Semester 1, 2, 3.

**Table 2:** Infant response to caregiver vocalisation by time, group, parent's gender (mother vs. father) and acoustic characteristics (parentese vs. other speech) in home movies from infants who will later develop autism (N = 14) versus typically developing infants (N = 14).

Variable	All infant responses	Toward people	Toward object	Receptive responses	Expressive responses	Exploratory responses	Inter-subjective responses	Active responses
Sem 2 vs. Sem 1	↗ β = 0.52 SE = 0.07351 (p<10^−11^)	↗ β = 0.34 SE = 0.07171 (p<10^−5^)	↗ β = 0.36 SE = 0.11302 (p = .001)	β = −0.02 SE = 0.10564 (ns)	β = −0.01 SE = 0.0857 (ns)	β = 0.23 SE = 0.1447 (ns)	↗ β = 1.39 SE = 0.2004 (p<10^−11^)	↗ β = 0.53 SE = 0.2020 (p = .007)
Sem 3 vs. Sem 2	β = 0.01 SE = 0.0789 (ns)	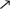 β = −0,21 SE = 0.0751 (p = .004)	↗ β = 0.40 SE = 0.103 (p<10^−4^)	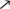 β = −0.84 SE = 0.14305 (p<10^−8^)	β = −0.15 SE = 0.0942 (ns)	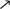 β = −0.39 SE = 0.1574 (p = .01)	↗ β = 0.54 SE = 0.1299 (p<10^−4^)	β = 0.17 SE = 0.17337 (ns)
AD vs. TD	β = −0.48 SE = 0.1510AD<TD (p = .001)	β = −0.39 SE = 0.1694 AD<TD (p = .01)	β = −0.13 SE = 0.2336 (ns)	β = −0.18 SE = 0.2789 (ns)	β = −0.40 SE = 0.2325 (ns)	β = 0.28 SE = 0.2382 (ns)	β = −0.87 SE = 0.3161 AD<TD (p = .005)	β = 0.03 SE = 0.3179 (ns)
Mother vs. Father	β = 0.08 SE = 0.0665 (ns)	β = 0.11 SE = 0.0643 (ns)	β = −0.07 SE = 0.0928 (ns)	β = −0.11 SE = 0.1053 (ns)	β = 0.23 SE = 0.0801 M>F (p = .004)	β = 0.16 SE = 0.1296 (ns)	β = −0.24 SE = 0.1237 M<F (p = .04)	β = 0.29 SE = 0.1614(ns)
Parentese vs. Other speech	β = 0.16 SE = 0.0703 P^ese^>OS (p = .02)	β = 0.17 SE = 0.0672 P^ese^>OS (p = .007)	β = −0.07 SE = 0.1004 (ns)	β = 0.52 SE = 0.1024 P^ese^>OS (p = 10^−6^)	β = 0.09 SE = 0.0803 (ns)	β = −0.10 SE = 0.1373 (ns)	β = −0.23 SE = 0.1453 (ns)	β = −0.22 SE = 0.1682 (ns)

M = mother; F = father; P^ese^ = Parentese; OS = other speech; AD = autism disorder; TD = typical development; SE = Standard Error; ns = non significant.

Although mothers vs. fathers did not differ in the number of responses they induced in their infant, mothers appeared to be significantly associated with infant expressive responses, whereas fathers were significantly associated with infant intersubjective behaviors (table 1). In particular, post-hoc analysis conducted on the AD group showed a significant association between fathers' vocalizations and both the intersubjective responses (β = 1.038; std error = 0.3702; p = .005) and active behavior (seeking people) during the third semester (β = −0.823; std error = 0.3676; p = .02).

Finally, as opposed to other speech, parentese (produced by the mother or the father) was also significantly associated with infant responses as a whole, infant responses involving orientation towards people and infant receptive responses (table 1). In particular, post-hoc analyses conducted on the AD group showed that parentese addressed to infants who will later develop autism was significantly associated with infant responses involving orientation towards people (β = 0.228; std error = 0.10188; p = .02) and infant receptive behaviours (β = 0.51610; std error = 0.15245; p = .0007); we also found that parentese addressed to infants who will later develop autism tended to be associated with infant expressive behaviours (e.g., vocal response) (β = 0.2401; std error = 0.1242; p = 0.05). When considering parentese facilitation of infant responses by semester, we found that parentese facilitation of infant response towards people (β = 0.6027; std error = 0.2150; p = 0.005) and infant receptive behaviours (β = 0.7640; std error = 0.2926; p = 0.009) appears as early as the first six months of life for infants with AD.

## Discussion

The use of engineering methods related to social signal processing allowed a focus on dynamic parent↔infant interaction instead of single behaviors of the baby or parent. It allowed us: (i) to focus on antecedents and consequences of interactive behaviours; (ii) to note significant sequences that could prompt or inhibit social interaction in a naturalistic way; (iii) to specifically study acoustic components of prosody, such as parentese, a key moderator of early interaction [8]. Our results regarding the facilitating effects of parentese on TD are concordant with the literature in which parentese is known to capture an infant's attention and elicit an infant's engagement. Concordant with this definition, in our study, parentese was significantly associated with “receptive” responses (looking at people, smiling at people, enjoying being with people and syntony). We discuss our results separately with regard to our initial hypotheses on children with AD, Dean Falk's theory of prelinguistic evolution in early hominins, and possible therapeutic implications in the early treatment of autism.

### Are the current results supporting our hypotheses?

We first hypothesised that parentese towards children with AD should decrease over time and that this decrease will likely be greater than that observed in parentese towards TD children. The results presented here did not support H1. We can interpret this result with at least three alternative explanations: (i) the 6-month time windows we used are too long to measure any differences in slope; (ii) TD infants are so interactive [15] that their parents do not need, after S2, to use parentese for successful interactions; or (iii) parents of AD infants use more parentese because they sense the on-going pathologic process. This third hypothesis is congruent with our previous results of an increasing of Regulation Up among CGs of infants with autism [15], as Regulation Up is “full” of parentese (figure 2B). A TD twin case study also has shown that a twin's mother used more parentese with the twin who was less reactive [30]. By suggesting that parents feel the pathological process ongoing, we want to guard against the idea that the parenting behaviors are impaired and cause autism. In fact, when parents respond to their infant they behave as parents of TD infant [15]. Rather, we suggest that they are some sort of reaction to early sign that are implicitly perceived by the parents. Although H1 is not supported, the lack of reciprocity from the AD infant could modify interaction dynamics, however, as we found that AD fathers were significantly more involved in interactions than TD fathers, particularly after one year. The importance of reciprocity and synchrony in early parent-infant interaction [14] was already supported in developmental studies of premature babies [31], in cases of parents being stressed during pregnancy [32] and in cases of parents with a previous history of a child with AD [33]. In the current study, parents, particularly fathers, did not receive any diagnosis but changed their behaviour to adapt to the lack of reciprocity. This behavioural change will be called father's involvement in interacting with infants, hereafter.

The second hypothesis (H2) proposed that AD infants should be capable of interacting with and responding to parentese from a very early age. The results presented here support H2, as we found that parentese (produced by the mother or the father) appeared to be significantly associated with infant responses as a whole and with infant responses involving orientation towards people. This was also the case for infants who will later develop AD. This finding may have promising therapeutic implications (see below).

### Implication for Dean Falk's theory regarding pre-linguistic evolution in early hominins

**Figure 3:**
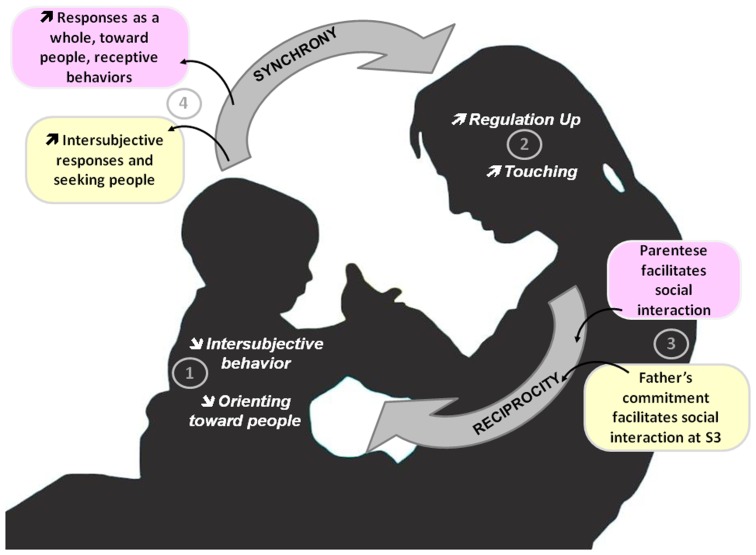
Infants who will later develop autism: pathologic trajectory of infant's behaviours and changes in parents' stimulation to adapt to their infant. In this figure, we summarised early interaction between infants who will subsequently develop autism and their parents. Infants show less intersubjective behaviours and orienting towards people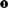. Parents adapt their behaviour by using more regulation up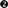 and touching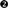 [13]. Regulation Up/Down is defined as caregiver vocalisation that modulates the child's arousal and mood, to either excite (reg-up) or calm (reg-down). Regulation up is full of parentese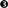, and this specific prosody appears to be significantly associated with the overall level of infants' responses, specifically infants' responses to parental vocalizations involving orientation towards people and receptive behaviours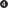. At the third semester (S3), compared to typically developing children, fathers of infants who will later develop autism appear to commit themselves more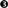 and the vocalisations of fathers of children with AD are significantly associated with infant's intersubjective responses and seeking people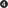.

Falk's theory regarding pre-linguistic evolution in early hominins is based on a comparison of mother-infant gestural and vocal interactions in chimpanzees and humans [34]. She noted that pre-linguistic vocal substrates that had prosodic features similar to contemporary parentese evolved with the trend regarding enlarging brains in late australopithecines/early Homo. This progressively increased the difficulty of parturition, causing a selective shift towards females that gave birth to relatively undeveloped neonates. In contrast to chimps, which favour physical contact with their infants that are mature enough to grasp his/her mother, Falk hypothesised that in humans, (i) mothers adopted new foraging strategies that entailed maternal silencing, reassuring, and controlling of the behaviurs of physically removed infants, (ii) the meanings of certain utterances (words) became conventionalised as mothers increasingly used prosodic and gestural markings to encourage juveniles to behave and obey. This hypothesis is based on the premises that hominin mothers that attended vigilantly to infants were strongly selected for and that such mothers had genetically based potentials for consciously modifying vocalizations and gestures to control infants, both of which receive support from the literature [19], [35]–[37]. Regarding human early interaction, this theory proposes touching, grasping, and skin-to-skin contact as old evolutionary moderators and parentese, gesturing, and infant crying as recent evolutionary moderators. Parents of infants who will develop AD change their interactive pattern of behaviour by recruiting both moderators (touching [15], as well as parentese and father involvement in interacting with infants [current results]) for interacting with their infants. These behaviours were found to be adaptive, and thus, the findings support Falk's theory. Although father involvement in interacting with infants is not discussed *per se* in Falk's theory, triadic interaction (involving baby, mother and father) may be specific to human social and family behaviors [38]. Although this field has been scarcely investigated, recent studies support the role of father in early interaction both at behavioural [39] and biological levels [40]. Figure 3 summarises, in infants who will later develop autism, the pathologic trajectory of infant behaviors and the changes in CG stimulations to adapt to their infant.

### Clinical implication for early treatment of autism

To our knowledge, the current study is the first to suggest that parentese is significantly associated with infant responses towards people in infants who later develop AD. For TD children, parentese seems to mediate (and also reflect) an interactive loop between the infant and his/her caregiver in which each partner's response may increase, in turn, the stimulation by the other partner. Moreover, this interactive loop (at the behavioural level) is underpinned by the emotional charge (at the affective level) and impacts attention, learning and the construction of intersubjective tools, such as joint attention and communicative skills (at the cognitive level) [18], [19]. Direct evidence for the interaction of cognitive and social levels is offered by Kuhl's finding that infant's learning of the phonetic properties of a language requires interaction with a live linguistic partner [41]. Audio-visual input alone is not sufficient. However, we were unable to find any study comparing interest in parentese versus other speech in children with autism. We know that (i) children with AD can process some aspects of human voices [42], although they display no specific cortical activation in response to human voices [43]; (ii) they do not show the expected preference for their mother's speech [44]; (iii) joint attention and immediate imitation appear to be important for setting the stage for early language acquisition in AD while representational skills (toy play, deferred attention) contribute to the expansion of communication skills [45]; (iv) also, children with AD fail to orient to naturally occurring social stimuli such as name called and hand clapping [46].

The results of the current study support the view that parentese, an early naturally occurring social stimulus, might provide useful feedback during social interaction sessions, particularly for very young children. Preliminary data support this view: (i) social and linguistic processing of speech in preschool children with autism could be correlated with behavioral and electrophysiological measures [47]; (ii) predictive correlations between attention to child directed speech (which is generally rich in parentese) and receptive language abilities were found in ASD [48], [49]; (iii) young ASD children showed less sustained attention to child directed speech performed by an actor compared to typically developing peers matched for chronological age. They did not, however, look less overall to child directed speech stimuli than typically developing peers matched for language age [50]; (iv) in a single case exploratory study, Laznik et al. observed HM sequences in which a withdrawn infant who will later develop autism suddenly appears joyful when the parent implements a vocal expression using parentese [27]. We now aim to explore the use of a parentese biofeedback loop based on our algorithm [21] to improve the emotional balance of CG prosody in the context of therapeutic interactive sessions (FP7 MICHELANGELO Project) and to assess whether parentese-like prosody is associated with better social interaction.

### Limits and strengths of this study

The first limitation is the sample size. Because we used rigorous statistical methods that took into account the random subject effect and autocorrelation, and because scenes were highly variable for a given infant (due to the great variability among scenes), some strong tendencies did not reach statistical significance. A larger sample would most likely have allowed us more analysable and/or significant results. Second, HM are not standardised, and analyses are retrospective from the time of positive diagnosis. Current research prefers prospective follow-up of high risk samples (e.g., siblings of AD children) with experimental procedures to assess early infant-parent interaction despite the fact that parents are aware of the risk. Recently, the British Autism Study of Infants' Siblings reported that early dyadic interaction between at-risk infants and their parents was associated with later diagnosis of autism [51]. This result is consistent with ours. Third, despite previous works with another pathologic group (infants who will develop intellectual disability without symptoms of AD), we were not able to include HM from this group in the current study, as the much lower occurrence of interaction, CG vocalisation and infant meta-behaviors prevented any statistical analysis [15]. However, to explore whether the severity of autism or comorbid ID significantly biased the current results, we performed a GLMM analysis with semester, speaker, type of speech, IQ and CARS score as the independent variables and infant response following a parental vocalization as the dependent variable. IQ and CARS scores showed no significant association with infant responses (data not shown). Finally, we modelled parent-child interactions as synchronic CGxInfant events using an approximation: CG vocalization followed by an infant response in a 3-second time window. However, this model is not similar to naturalistic observations, which take into account longer lasting periods of time and more continuous series of events (e.g., latency response measures, cross recurrence analysis, and slope analysis) [52], [53]. Strengths of the study include the spontaneous adaptation to the pathologic process that is observable in HM compared to the study of infant-parent interaction in the case of studies with ASD siblings [33] or to more experimental settings. Indeed, Wan et al. [33] suggested that infant siblings at risk of autism were exposed to a more directive parental interactive style relatively early in infancy. Second, the use of an automatic detector of acoustic components in prosody related to emotion [21] helped us in distinguishing infant-directed speech with parentese prosody versus infant-directed speech without such prosody. Third, we were able to distinguish between mother versus father, which allowed the assessment of both dyadic and triadic dynamics [54]. Fourth, despite its size, the group of patients was quite homogeneous because of rigorous exclusion criteria used in the Pisa HM data base.

## Conclusion

Parents of infants who will develop autism change their interactive pattern of behaviour, increasing both parentese prosody and father's involvement in interacting with infants; both are significantly associated with infant's social responses. We stress the possible therapeutic implications of these findings.

## Supporting Information

Table S1 **Clinical characteristics of each infant included in the AD group (N = 14).**
(DOC)

Table S2 **Number of scenes, parental vocalizations, maternal vocalizations, paternal vocalizations and infant responses per infant.**
(DOC)

Annex S1 **Infant's and caregiver's behaviors and meta-behviors from the infant caregiver behavior scale (ICSB).**
(DOC)
