# Supplementary material for: Do Parentese Prosody and Fathers' Involvement in Interacting Facilitate Social Interaction in Infants Who Later Develop Autism?
Source: PLoS One. 2013 May 1;8(5):e61402. doi: 10.1371/journal.pone.0061402 (PMC3641085; doi:10.1371/journal.pone.0061402)
Supplement: Table S1 — Clinical characteristics of each infant included in the AD group (N = 14). (DOC) [file pone.0061402.s001.doc]

| **Table S1: Clinical characteristics of each infant included in the AD group (N=14)** | | | | | | |
| --- | --- | --- | --- | --- | --- | --- |
| We studied home movies (HM) from the first 18 months of life of two groups of children. The first group is composed of 15 children (M/F: 11/4) with a diagnosis of Autistic Disorder (AD) according to the DSM-IV criteria. In addition, we used the ADI-R for diagnosis and the Childhood Autism Rating Scale (CARS) to assess the severity of autistic symptoms. All cases with a CARS total-scores below 30 and with Pervasive Developmental Disorders Not Otherwise Specified were excluded. The absence of an identified genetic or metabolic disorder and of a severe sensory or motor impairment were verified for all children. The AD group is composed of children with an early onset autism without any history of regression: it means that all children displayed, from the beginning, the autistic symptoms constellation rated through the Behavioral Summarized Evaluation applied to the home movies of the first year of life (see Muratori et al., 2010). The children were recruited among those referred from multiple community sources to the the Scientific Institute “IRCCS Stella Maris”, a suburban university hospital providing tertiary care to patients of all socioeconomic levels. A control group (TD), composed of 15 children (M/F: 9/6), was recruited among children attending a local kindergarten: these children have a history of typical development confirmed by a non clinical score at the Child Behavior Check List (Achenbach, 2000). Inclusion criteria for all groups was the presence of HM without any editing by the parents and running for a minimum of 10 minutes for each of the three period analyzed in the study. | | | | | | |
|  | Sex | IQ* | CARS | ADI-R social | ADI-R communication | ADI-R stereotyped behaviour |
| AD1 | F | 79 | 47 | 10 | 13 | 2 |
| AD 2 | M | 63 | 31.5 | 9 | 18 | 2 |
| AD 3 | M | 96 | 38 | 24 | 11 | 6 |
| AD 4 | M | 83 | 35.5 | 7 | 9 | 6 |
| AD 5 | F | 45 | 48.0 | 10 | 13 | 6 |
| AD 6 | F | 67 | 38.5 | 10 | 15 | 8 |
| AD 7 | M | 75 | 31.0 | 22 | 7 | 2 |
| AD 8 | M | 63 | 31.5 | 19 | 10 | 6 |
| AD 9 | M | 67 | 42.0 | 8 | 13 | 4 |
| AD 10 | M | 62 | 43.5 | 14 | 21 | 10 |
| AD 11 | F | 62 | 32.0 | 10 | 12 | 5 |
| AD 12 | M | 115 | 32.0 | 9 | 10 | 7 |
| AD 13 | M | 68 | 36.0 | 13 | 11 | 4 |
| AD 14 | M | 75 | 33.0 | 21 | 18 | 7 |
| AD: Autism Disorder; M: Male; F: Female; IQ: Intellectual Quotient; CARS: Children Autism Rating Scale; ADI-R: Autistic Diagnosis Interview-Revised.  *IQ in children with AD was based on the Griffiths Mental Developmental Scale or the Wechsler Intelligence Scale. | | | | | | |
