# Supplementary material for: Do Parentese Prosody and Fathers' Involvement in Interacting Facilitate Social Interaction in Infants Who Later Develop Autism?
Source: PLoS One. 2013 May 1;8(5):e61402. doi: 10.1371/journal.pone.0061402 (PMC3641085; doi:10.1371/journal.pone.0061402)
Supplement: Table S2 — Number of scenes, parental vocalizations, maternal vocalizations, paternal vocalizations and infant responses per infant. (DOC) [file pone.0061402.s002.doc]

| Table S2: Number of scenes, parental vocalizations, maternal vocalizations, paternal vocalizations and infant responses per infant. | | | | | |
| --- | --- | --- | --- | --- | --- |
| Infants | Scenes (N) | Parental vocalizations (N) | Mother’s vocalizations (N) | Father’s vocalizations (N) | Infant responses (N) |
| *Infant who will develop autism* | | | | | |
| AD1 | 10 | 77 | 39 | 38 | 50 |
| AD 2 | 13 | 104 | 78 | 26 | 37 |
| AD 3 | 6 | 87 | 62 | 25 | 46 |
| AD 4 | 18 | 267 | 190 | 77 | 173 |
| AD 5 | 14 | 478 | 167 | 311 | 310 |
| AD 6 | 10 | 88 | 55 | 33 | 34 |
| AD 7 | 13 | 290 | 69 | 221 | 109 |
| AD 8 | 18 | 332 | 225 | 107 | 230 |
| AD 9 | 11 | 244 | 160 | 84 | 156 |
| AD 10 | 4 | 27 | 12 | 15 | 10 |
| AD 11 | 15 | 110 | 75 | 35 | 69 |
| AD 12 | 9 | 133 | 61 | 72 | 79 |
| AD 13 | 3 | 4 | 0 | 4 | 2 |
| AD 14 | 6 | 66 | 9 | 57 | 47 |
| AD total | 150 | 2247 | 1202 | 1105 | 1352 |
| *Infant showing typical development* | | | | | |
| TD 1 | 10 | 238 | 124 | 114 | 164 |
| TD 2 | 11 | 155 | 86 | 69 | 113 |
| TD 3 | 9 | 127 | 127 | 0 | 90 |
| TD 4 | 17 | 250 | 140 | 110 | 168 |
| TD 5 | 15 | 368 | 151 | 217 | 241 |
| TD 6 | 11 | 220 | 191 | 29 | 157 |
| TD 7 | 12 | 177 | 121 | 56 | 139 |
| TD 8 | 12 | 160 | 89 | 71 | 107 |
| TD 9 | 13 | 254 | 156 | 98 | 147 |
| TD 10 | 11 | 49 | 38 | 11 | 30 |
| TD 11 | 15 | 302 | 177 | 125 | 197 |
| TD 12 | 8 | 211 | 179 | 32 | 139 |
| D 13 | 17 | 341 | 174 | 167 | 217 |
| TD 14 | 2 | 37 | 28 | 9 | 23 |
| TD total | 163 | 2889 | 1781 | 1108 | 1932 |
