# Supplementary material for: Do Parentese Prosody and Fathers' Involvement in Interacting Facilitate Social Interaction in Infants Who Later Develop Autism?
Source: PLoS One. 2013 May 1;8(5):e61402. doi: 10.1371/journal.pone.0061402 (PMC3641085; doi:10.1371/journal.pone.0061402)
Supplement: Annex S1 — Infant's and caregiver's behaviors and meta-behviors from the infant caregiver behavior scale (ICSB). (DOC) [file pone.0061402.s003.doc]

| Annex 1. Infant’s and caregiver’s behaviors and meta-behviors from the infant caregiver behavior scale (ICSB) | | |
| --- | --- | --- |
| **Meta-behavior** | **Item Behavior** | Glossary |
| *Child Behaviors* | | |
| Behavior with object | Orienting toward object | The child directs his/her gaze towards a source of new sensory stimulation coming from an object |
|  | Gaze Following an object | The child shifts his/her gaze to follow the trajectory of an object. |
|  | Explorative activity with object | The child touches something by hands, mouth or other sensory-motor actions, to find out what it feels like. |
|  | Looking at object/around | The child directs his/her eyes towards an object, or simply looks around. |
|  | Smiling at object | The child intentionally smiles at object. |
|  | Enjoying with object | The child finds pleasure and satisfaction experiencing a physical or visual contact with an object. |
|  | Seeking contact with object | The child employs spontaneous and intentional movements to reach contact with an object. |
| Vocalizations | Simple Vocalisation | The child produces sounds towards people or objects. |
|  | Crying | The child starts crying after a specific/non specific event. |
| Orienting toward people | Orienting toward people | The child directs his/her gaze towards a source of new sensory stimulation coming from a people |
|  | Gaze Following a person | The child shifts his/her gaze to follow the trajectory of another person. |
|  | Explorative activity with person | The child touches a person to find out what it feels like (by hands, mouth or other sensory-motor actions). |
| Receptive to people | Looking at people | The child directs his/her eyes towards a human face. |
|  | Smiling at people | The child intentionally smiles at a person. |
|  | Enjoying with person | The child finds pleasure and satisfaction experiencing a physical or visual contact with a person. |
|  | Sintony | The child shows signs of congruous expressions to affective solicitations, to the other’s mood. |
| Seeking people | Seeking contact with person | The child employs spontaneous and intentional movements to reach contact with a person. |
|  | Soliciting | The child displays a vocal or tactile action to attract the partner’s attention or to elicit another response. |
| Inter-subjective behavior | Anticipation of other’s intention | The child makes anticipatory movements predicting the other’s action. |
|  | Communicative gestures | The child displays use of social gestures. |
|  | Referential gaze | The child shifts his/her gaze towards the caregiver to look for consultation in a specific situation. |
|  | Gaze following gaze | The child shifts his/her gaze to follow the gaze of another person. |
|  | Accept Invitation | The child’s behavior is attuned to the person’s solicitation within 3 seconds. |
|  | Orienting to name prompt | The child assumes a gaze direction towards the person who calls him/her by the name. |
|  | Imitation | The child repeats, after a short delay, another person’s action. |
|  | Pointing comprehensive/ declarative/requestive | The child a) shifts his/her gaze towards the direction pointed by a person; b) points something in order to share an experience; c) in order to obtain an object. |
|  | Maintaining social engagement | The child takes up an active role within a two-way interaction in order to keep the other person involved. The child interacts, vocalises and maintains turn taking. |
|  | Meaningful Vocalisation | The child intentionally produces sounds with a stable semantic meaning |
| ***Caregiver’s Behaviors*** | | |
| Reg-up/down | Regulation up/down | modulates the child’s arousal and mood, to either excite (reg-up) or calm (reg-down). |
| Touching | touching | stimulates the child requesting attention by touching him/her. |
| Vocalization | vocalizing/naming/behavior request | stimulates the child requesting attention by vocalizing, naming |
| Gesturing-showing | gesturing/showing object | stimulates the child requesting attention by gesturing or showing him object |
